# Whole exome sequencing enables the correct diagnosis of Frank–Ter Haar syndrome in a Saudi family

**DOI:** 10.18699/vjgb-24-37

**Published:** 2024-06

**Authors:** Y.N. Khan, M. Imad A.M. Mahmud, N. Othman, H.M. Radzuan, S. Basit

**Affiliations:** Department of Basic Medical Sciences, Faculty of Medicine, International Islamic University Malaysia; Department of Basic Medical Sciences, Faculty of Medicine, International Islamic University Malaysia; Department of Basic Medical Sciences, Faculty of Medicine, International Islamic University Malaysia; Department of Basic Medical Sciences, Faculty of Medicine, International Islamic University Malaysia; Department of Biochemistry and Molecular Medicine, College of Medicine, Taibah University Al Madinah Al Munawara, Saudi Arabia Center for Genetics and Inherited Diseases, Taibah University Al Madinah Al Munawara, Saudi Arabia

**Keywords:** exome sequencing, mutation, SH3PXD2B gene, Frank–Ter Haar syndrome, секвенирование экзома, мутация, ген SH3PXD2B, синдром Франка–Тер Хаара

## Abstract

Frank–Ter Haar syndrome (FTHS) is a rare genetic hereditary autosomal recessive disorder characterized by defective malformation of cardiovascular, craniofacial, and skeletal system. Mutations in the SH3PXD2B gene are a common cause in the development of FTHS. We recruited a family with two affected individuals (3-year-old female and 2-month-old male infant) having bilateral clubfoot. Family pedigree shows an autosomal recessive mode of inheritance. DNA was extracted from the blood samples of six members of the family. Whole exome sequencing was done for the two affected individuals and the variant was validated in the whole family by using Sanger sequencing approach. Whole exome sequencing (WES) data analysis identified a rare homozygous variant (c.280C>G; p.R94G) in the SH3PXD2B gene, and Sanger sequencing showed that the same variant perfectly segregates with the phenotype in the pedigree. Moreover, the variant is predicted to be damaging and deleterious by several computation tools. Revisiting the family members for detailed clinical analysis, we diagnosed the patients as having the typical phenotype of FTHS. This study enabled us to correctly diagnose the cases of FTHS in a family initially recruited for having bilateral clubfoot by using WES. Moreover, this study identified a novel homozygous missense variant (c.280C>G; p.R94G) in (NM_001308175.2) the SH3PXD2B gene as a causative variant for autosomal recessive FTHS. This finding supports the evidence that homozygous mutations in the SH3PXD2B gene are the main cause in the development of FTHS.

## Abstract (ru)

Синдром Франка–Тер Хаара (Frank–Ter Haar syndrome, FTHS) – редкое генетическое заболевание с аутосомно-рецессивным типом наследования, характеризующееся аномалиями развития сердечно-сосудистой системы, костей лицевого черепа и скелета. Наиболее распространенной причиной развития данного синдрома являются мутации в гене SH3PXD2B. Для исследования была выбрана семья, в которой двое детей (трехлетняя девочка и двухмесячный мальчик) страдали двусторонней косолапостью. В семейной родословной указывался аутосомно-рецессивный тип наследования. Из крови шести членов семьи мы выделили образцы ДНК. Для упомянутых двоих детей было проведено полное секвенирование экзома, а секвенированием по Сэнгеру подтверждено наличие мутантного варианта у всех членов семьи. По результатам анализа данных полноэкзомного секвенирования (WES) была выявлена редкая гомозиготная мутация (c.280C>G; p.R94G) в гене SH3PXD2B. Секвенирование по Сэнгеру показало, что эта мутация идеально сегрегирует с указанным фенотипом в родословной. Более того, при использовании ряда инструментальных средств получены данные, предсказывающие вредность и опасность этой мутации. При повторном посещении членов семьи с целью проведения развернутого клинического анализа было установлено, что фенотип двоих детей, страдавших двусторонней косолапостью, характерен для больных с синдромом FTHS. Таким образом, исследование позволило безошибочно диагностировать синдром FTHS в семье, первоначально выбранной в связи с двусторонней косолапостью у ее членов, с помощью WES. Более того, наше исследование показало, что причиной развития синдрома FTHS с аутосомно-рецессивным типом наследования была вновь выявленная гомозиготная миссенсмутация (c.280C>G; p.R94G) в гене (NM_001308175.2) SH3PXD2B. Это служит дополнительным подтверждением существующих данных о том, что гомозиготные мутации в гене SH3PXD2B являются основной причиной развития синдрома FTHS

## Introduction

Frank–Ter Haar syndrome (FTHS) is a rare genetic hereditary autosomal recessive disorder characterized by cranial deformities like wide fontanelle and enlarged forehead, facial deformities such as small chin and full cheeks, ocular anomalies, namely exophthalmos, enlarged cornea with or without glaucoma and hypertelorism, protruded ear auricles, cardiovascular and skeletal deformities including a long coccyx bone with an overlying skin fold (Mass et al., 2004). Clinical features and genetic relations of the syndrome were first described by Frank et al. in a Dutch family in 1973 (Frank et al., 1973). Nine years later, Ter Haar et al. confirmed that the phenotype is inherited in an autosomal recessive manner (ter Har et al., 1982). Hence the name of the phenotype – Frank–Ter Haar syndrome.

Genetic studies suggested that mutation in the SH3PXD2B gene is a common cause in the development of FTHS. A study on 13 homozygously affected families mapped out and revealed gour different intronic mutations with two complete deletions in the SH3PXD2B gene (Iqbal et al., 2010; Massadeh et al., 2022). A knock out study showed that a deficient protein TKS4 encoded by the SH3PXD2B gene presents similar morphological features such as craniofacial, musculoskeletal, cardiovascular, and ocular anomalies (Iqbal et al., 2010). A literature review by Durand B. et al. in 2020 showed that 40 patients manifesting clinical features similar to FTHS have been reported worldwide, half of them were carrying mutations in SH3PXD2B (Durand et al., 2020).

Whole exome sequencing (WES) has revolutionized the modern era of clinical diagnosis, especially the diseases with variable phenotypic presentations and of multiorgan involvement. Whole exome sequencing allows the diagnosis of monogenic diseases and is recommended by the American College of Medical Genetics and Genomics (ACMG) as a first-line testing option to detect mutations causing genetic disorders presenting one or more congenital abnormalities and development delays, also ascertaining potential risks in individuals prior to disease manifestation, thereby avoiding unnecessary diagnostic tests (Manickam et al., 2021). One study accurately established the clinical diagnosis of Cohen syndrome when genomic analysis on DNA samples of affected and unaffected individuals was performed; otherwise, the diagnosis would have been impossible to make because of the different clinical presentations of the same disease in the affected family members (Hashmi et al., 2020). García-Aznar et al. reported a female patient having features suggestive of Soto syndrome and initial genetic analysis did not reveal a mutation in the pathogenic gene but whole exome sequencing of all the genes showed a frameshift variant in the AMER1 gene causing the phenotype of osteopathia striata with cranial sclerosis, which was later confirmed upon doing retrospective clinical and instrumental examination (García-Aznar et al., 2021). Hence, the role of the whole exome sequencing is crucially important in diseases with non-specific clinical presentations. Furthermore, exome sequencing carries a positive impact on management of the affected individuals and genetic counseling of their family members. A case report of a patient with severe transfusiondependent anemia that was clinically diagnosed as Diamond– Blackfan anemia (DBA), but WES analysis finally revealed the condition as a variant of hereditary hemolytic anemia. Thus, the child was successfully managed with splenectomy, which ultimately reduced his blood transfusion dependency (Khurana et al., 2018).

Here we report a family of 6 members, where two children having bilateral clubfoot were studied to identify the genetic defects underlying the clubfoot phenotype. WES identified a pathogenic variant in the SH3PXD2B gene. Clinical reexamination revealed additional morphological features in the patients, establishing the diagnosis as FTHS.

## Methods

A single four-generation family with 2 affected individuals was phenotypically and genetically analyzed. The family pedigree shown in the Figure was drawn to assess the pattern of inheritance of this disorder. Ethical review committee date 20-09-2020 Study ID: 036-1441 of the Taibah University, Medina, Kingdom of Saudi Arabia approved the research study. Parents of the affected individuals signed the written informed consent after understanding the aims of the study, which were explained in their local (Arabic) language

**Fig. 1.**
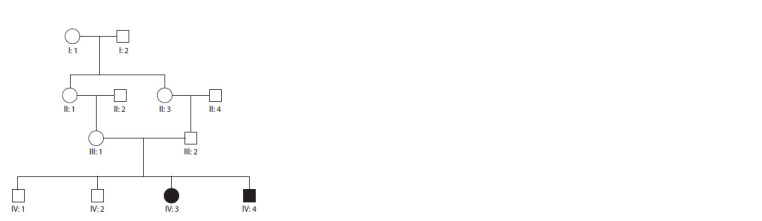
Family pedigree shows consanguinity, carriers, and affected individuals. The pedigree depicts an autosomal recessive mode of inheritance for this variant mutation. The female and male individuals are represented with circle and square symbols respectively. Filled symbols signify homozygous individuals for the missense variant (c.280C>G) in SH3PXD2B.

Genomic study (DNA extraction). Blood samples were collected from the parents (III:1 and III:2), two unaffected healthy sibs (IV:1 and IV:2) and two affected individuals (IV:3 and IV:4) (see the Figure). Genomic DNA was extracted by using the QIAmp DNA micro kit (Hilden, Germany). DNA quantity and quality was assessed by using a Nano Drop TM spectrophotometer

Next Generation Sequencing (NGS) methods. After confirming the standard DNA quality and quantity, whole exome sequencing was performed on the affected individuals (IV:3 and IV:4) using the Illumina HiSeq 2500 platform (Illumina, San Diego, CA, USA). The SureSelect Target Enrichment Kit v6 was used to prepare the libraries as elaborated in earlier studies (Rafiullah et al., 2022; Ullah et al., 2022). Sequencing data coverage was 30x and sequencing data depth was 100x. Standard filtration steps were followed to analyze VCF (variant calling files) of the two affected individuals, which were uploaded by using the online Illumina Base Space analysis tool (https://basespace.illumina.com). As shown in the family pedigree (see the Figure), due to an autosomal recessive pattern of inheritance with consanguineous marriage in the family, only two affected individuals having homozygous and heterozygous variants were filtered for the analysis.

Sanger sequencing for validation and segregation analysis. Variant-specific primers were designed for the prioritized variant after exome filtration. Ensembl genome browser (https://m.ensembl.org) was used to download the exonic sequence for the specific gene. Primer 3 software (http://primer3. ut.ee) was used to design the specific primers for identified variants with 30x sequencing data coverage and 100x sequencing data depth. Purification of PCR-amplified DNA was achieved using the Marligen Biosciences kits (Ijamsville, MD, USA). Sanger sequencing was performed using the BigDye sequencing kit (Applied Biosystems, USA) as described earlier (Alluqmani, Basit, 2022; Ijaz et al., 2022). Alignment of the Sanger sequencing reads with reference sequences were obtained using BIOEDIT to confirm variant identity

In silico tools were used to calculate pathogenicity scores. Various in silico tools were used to calculate the pathogenicity scores including meta scores as well as individual scores of the variant by using BayesDel addAF (https:// fengbj-laboratory.org/BayesDel/BayesDel.html), MetaLR (https://www.ensembl.org/info/genome/variation/prediction/ protein_function.html), MetaSVM (http://cancergenome.nih. gov), and REVEL (https://blog.goldenhelix.com/annotateyour- varseq-projects-with-revel/engines). Moreover CADD, (https://asia.ensembl.org/info/genome/variation/prediction/ protein_function.html#CADD), DANN, FATHMM, LRT, Mutation assessor (http://fathmm.biocompute.org.uk/), MutationTaster (https://www.mutationtaster.org/), MutPred (http://mutpred.mutdb.org/), PolyPhen2 (http://genetics.bwh. Haarvard.edu/pph2/), PROVEAN (https://www.jcvi.org/ research/provean), and SIFT (https://www.merriam-webster. com/dictionary/sift) engines were also used to calculate individual pathogenicity scores.

## Results

Both affected individuals were referred to specialists in multiple disciplines such as pediatrician, cardiologist, ophthalmologist, orthopedic surgeon, pediatric neurologist and finally referred to a specialist in clinical genetics at the Maternity and Children Hospital, Al Madinah Al Munawara for further evaluation and care. Details of the clinical presentation of both cases (IV:3, IV:4) as documented by the specialists of different clinical departments at the Maternity and Children Hospital Al Madina Al Munawara are mentioned in Table 1

**Table 1.**
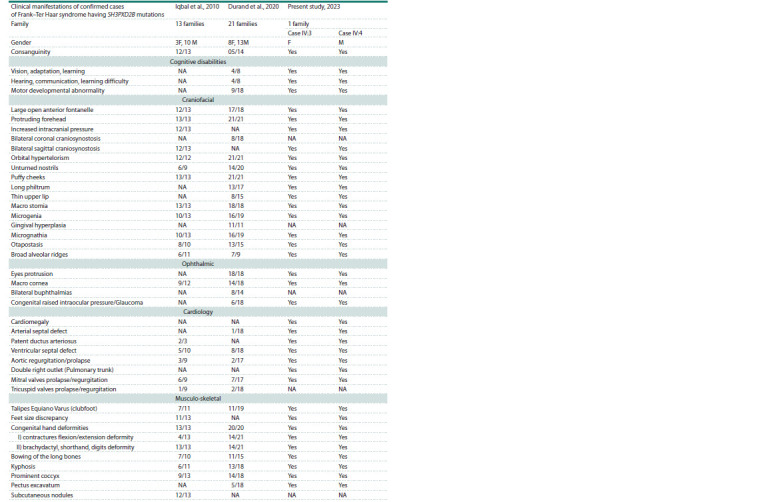
Comparison of the clinical manifestations of Frank–Ter Haar syndrome in family studies by Iqbal et al., 2010, and by Durand et al., 2020 Note. –/–, Number of families positive for mentioned clinical features/total number of families studied. “Yes” is for patients having the mentioned clinical features and “NA” indicates the not available or absence of the clinical features.

Potentially pathogenic missense mutation in SH3PXD2B in both patients

Sequencing reads were aligned to the reference genome and variants were annotated and prioritized based on the phenotype of the patients (IV:3 and IV:4). WES data failed to identify any pathogenic variant in the genes associated with clubfoot. All variants in the WES data were annotated, filtered, and prioritized for rare (minor allele frequency less than 0.001), homozygous or heterozygous, shared (common to both affected individuals) and potentially pathogenic variants (based on SIFT and PolyPhen2 scores). Variants in OBSL1 (NM_015311.3; c.4989+5G>A), SH3PXD2B (NM_001308175.2; c.280C>G; p.R94G), and MAN2B1 (NM_000528.4; c.2402dupG; p.S802fs*129) were initially prioritized.

Sanger sequencing validated and confirmed the autosomal recessive inheritance of the SH3PXD2B variant in the family

Primers were designed for all three variants that were amplified by polymerase chain reaction (PCR) in all available members III:1, III:2, IV:1, IV:2, IV:3, IV:4 of the family. Variants in OBSL1 (c.4989+5G>A) and MAN2B1 (c.2402dupG) were found not to segregate in the family, therefore, they were not considered for further analysis. A variant in SH3PXD2B (c.280C>G) perfectly segregates with the phenotype in the pedigree. Both parents and unaffected individuals are found to be heterozygous for the variant and both affected individuals are homozygous for it. Therefore, a rare (0 % gnomAD frequency) homozygous missense variant (c.280C>G; p.R94G) in the SH3PXD2B (NM_001308175.2) gene was considered as the most plausible candidate variant for the disease phenotype in this family. The variant is present in the exome data of both affected individuals (IV:3 and IV:4).

In silico analysis predicted the variant (c.280C>G) in SH3PXD2B to be potentially pathogenic

Most of the in silico engines including CADD, DANN, FATHMM, LRT, Mutation assessor, MutationTaster, MutPred, PolyPhen2, PROVEAN, and SIFT predicted the variant to be disease causing, damaging or pathogenic. Table 2 shows the score and prediction obtained after analyzing the variant with various in silico software. A very low frequency in gnomAD (PM2) and support from multiple lines of computational evidence (PolyPhen2, SIFT, CADD) (PP3), as well as segregation of the variant with the disease phenotype in the family support the hypothesis that this variant is an underlying cause of the phenotype in our case

**Table 2.**
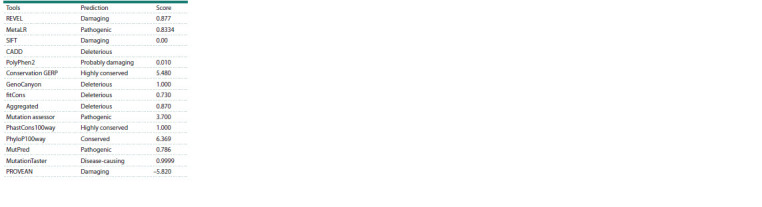
In silico analytical prediction of the potential pathogenicity of the missense variant (c.280C>G) in SH3PXD2B

## Discussion

Congenital inherited disorders such as FTHS have broad overlapping clinical presentations that often make them difficult and unlikely to be diagnosed. Biochemical laboratory tests do not even show any evidential clues for these disorders and the genes are only investigated for research purposes. Nextgeneration technologies such as whole exome sequencing are considerably affordable, and a preferable testing platform in situations where two or more than two affected individuals are found in a consanguineous marriage family (Alluqmani, Basit, 2022).

In this study, a consanguineous marriage family from Saudi Arabia having two affected individuals was investigated both clinically and genetically. The family was referred to the Center for Genetics and Inherited Diseases, Taibah University for the genetic diagnosis of clubfoot. Family members were registered, and WES was performed. Initially, genes associated with clubfoot (PITX1, TBX4, HOXA9, HOXD10, HOXD12, HOXD13, HOXA9, TPM1, TPM2, COL9A1, FLNB, CASP8, CASP10, UTX, CHD1, RIPPLY2, CAND2, WNT7) were screened for potential variants. However, WES data analysis failed to detect any potential pathogenic variant in clubfootassociated genes. Therefore, an unbiased and hypothesis-free approach was used to analyze WES data to filter and prioritize variants of interest. A potentially pathogenic variant in the SH3PXD2B gene was identified. Patients were recalled by the physician, and they were thoroughly re-examined. Clinical review of the affected individuals showed additional features of musculoskeletal deformity, cardiac, ophthalmic, craniofacial disorders, and cognitive disabilities. These clinical features helped us to classify our cases as FTHS (Iqbal et al., 2010). In this family, the affected individuals were also found to have cardiomegaly and a double pulmonary trunk, which were not reported previously. While gingival hyperplasia, buphthalmia, and subcutaneous nodules are the features commonly reported in such cases in the literature, these are not seen in our cases (Durand et al., 2020).

FTHS is primarily caused by mutation in the SH3PXD2B gene. This gene, located on 5q35.1 chromosome, encodes a 911-amino-acid protein, which has a phox homology (PX) domain, known as Tks4 (tyrosine kinase substrate with four SH3 domains) (Iqbal et al., 2010). This protein is involved in the formation of actin-rich membrane protrusions called podosomes, which coordinate pericellular proteolysis with cell migration and regulate proliferation, growth, and differentiation in the cells with extracellular matrix remodeling (Gimona et al., 2008). The gene mutation leads to the absence of Tks4 and thus embryonic fibroblasts decrease the formation of mature and functional podosomes; hence, they fail to degrade the extracellular matrix (Saeed et al., 2011). Filamin A protein is present in the podosome belt, and it needs to be cleaved by calpain for maintaining osteoclast motility during bone development (Marzia et al., 2006). Filamin A is also required for podosome rosette formation, proteolysis of the extracellular matrix mediated by podosomes in macrophages, and threedimensional mesenchymal cells build up, so mutation in the genes encoding for actin-rich membrane structures causes serious congenital anomalies of the heart, skeleton, and craniofacial region (Cejudo-Martin, Courtneidge, 2011). Newly published knockout studies proved that TKS4, once lost, can adversely affect the differentiation of different cell lineages and maturation processes, thus leading to the development of FTHS (László et al., 2022).

Hence, the ambiguous clinical presentation of FTHS is commonly seen due to overlapping features as the defect occurs during the differentiation of primordial germ layer development, which influences multiple organs and systems of the organism. Therefore, clinical use of genetic testing like WES is essential when a clinician encounters a case showing unclear clinical and/or laboratory presentation (Sharma, Nalepa, 2016).

Whole exome sequencing has played an important role in diagnosis of other diseases as well. A consanguineous Saudi family having five individuals with steroid resistant Nephrotic syndrome were examined by WES which identified a homozygous novel insertion mutation (c.6272_6273insT) in the PLCE1 gene (Hashmi et al., 2018). WES is also considered a useful time-saving practical diagnostic tool in the evaluation of patients with rare and complex hereditary disorders like episodic ataxia type 1. This diagnostic approach can hasten early therapeutic intervention strategies and directly affect patient care (Tacik et al., 2015).

## Conclusion

This study provides us with further evidence for the importance of validation of genetic variants involved in the development of the FTHS with the use of WES. Here we reported that the homozygous missense variant (c.280C>G; p.R94G) in the SH3PXD2B (NM_001308175.2) gene can be considered as the candidate variant resulting in autosomal recessive FTHS. This study covers the SH3PXD2B gene mutation spectrum, which might further reflect on the importance of properly correlating genotypes with phenotypes and provides support to the importance of genetic testing and analysis of the SH3PXD2B gene in the Kingdom of Saudi Arabia and probably certain other locations. This will also be beneficial in marriage counseling and planning of future pregnancies among FTHS carrier families.

## Conflict of interest

The authors declare no conflict of interest.
